# Differential routing and disposition of the long-chain saturated fatty acid palmitate in rodent vs human beta-cells

**DOI:** 10.1038/s41387-022-00199-y

**Published:** 2022-04-20

**Authors:** Patricia Thomas, Catherine Arden, Jenna Corcoran, Christian Hacker, Hannah J. Welters, Noel G. Morgan

**Affiliations:** 1 Institute of Metabolism and Systems Research, Birmingham Medical School, University of Birmingham, Birmingham, UK; 2 Biosciences Institute, The Medical School, Newcastle University, Newcastle upon Tyne, UK; 3 Department of Biosciences, University of Exeter, Geoffrey Pope Building, Exeter, UK; 4 Bioimaging Centre, College of Life and Environmental Sciences, University of Exeter, Exeter, UK; 5 Institute of Biomedical and Clinical Research, College of Medicine and Health, University of Exeter, Exeter, UK

**Keywords:** Organelles, Type 2 diabetes

## Abstract

**Background:**

Rodent and human β-cells are differentially susceptible to the “lipotoxic” effects of long-chain saturated fatty acids (LC-SFA) but the factors accounting for this are unclear. Here, we have studied the intracellular disposition of the LC-SFA palmitate in human vs rodent β–cells and present data that reveal new insights into the factors regulating β-cell lipotoxicity.

**Methods:**

The subcellular distribution of the LC-SFA palmitate was studied in rodent (INS-1E and INS-1 823/13 cells) and human (EndoC-βH1) β-cells using confocal fluorescence and electron microscopy (EM). Protein expression was assessed by Western blotting and cell viability, by vital dye staining.

**Results:**

Exposure of INS-1 cells to palmitate for 24 h led to loss of viability, whereas EndoC-βH1 cells remained viable even after 72 h of treatment with a high concentration (1 mM) of palmitate. Use of the fluorescent palmitate analogue BODIPY FL C_16_ revealed an early localisation of the LC-SFA to the Golgi apparatus in INS-1 cells and this correlated with distention of intracellular membranes, visualised under the EM. Despite this, the PERK-dependent ER stress pathway was not activated under these conditions. By contrast, BODIPY FL C_16_ did not accumulate in the Golgi apparatus in EndoC-βH1 cells but, rather, co-localised with the lipid droplet-associated protein, PLIN2, suggesting preferential routing into lipid droplets. When INS-1 cells were treated with a combination of palmitate plus oleate, the toxic effects of palmitate were attenuated and BODIPY FL C_16_ localised primarily with PLIN2 but not with a Golgi marker.

**Conclusion:**

In rodent β-cells, palmitate accumulates in the Golgi apparatus at early time points whereas, in EndoC- βH1 cells, it is routed preferentially into lipid droplets. This may account for the differential sensitivity of rodent vs human β-cells to “lipotoxicity” since manoeuvres leading to the incorporation of palmitate into lipid droplets is associated with the maintenance of cell viability in both cell types.

## Introduction

A reduction in functional β-cell mass is central to the pathogenesis of type 2 diabetes (T2D) [1], although the underlying mechanisms are unclear. In many populations, T2D appears to increase in parallel with rising nutritional status [2] and, in particular, there are suggestions that increased uptake of saturated fats may be specifically detrimental to β-cell health and viability [3]. Such evidence is supported by data revealing that rodent β-cells become compromised when exposed chronically to long-chain saturated fatty acids (LC-SFA) in vitro [3] in a process which, although not universally accepted [4] has been termed “lipotoxicity”.

In vivo, abnormal fatty acid storage and mobilisation are frequently associated with insulin resistance and this is understood to be an early manifestation of emerging T2D [5]. As a result, plasma FFA can become elevated (from approx. 0.1 mM to ->1 mM) [6] and their circulating profiles altered with increases in LC-SFA species, particularly palmitate (C16:0) and stearate (C18:0) [7]. This is important because, on the basis of work conducted in vitro, there is a firm consensus that chronic treatment of β-cells with LC-SFA can lead to dysfunction and the ultimate loss of viability [3, 8, 9]. However, the situation is complex since not all fatty acids exert similar actions and, in particular, long-chain monounsaturated fatty acids (LC-MUFA) such as oleate (C18:1) and palmitoleate (C16:1) are well tolerated by rat β-cells during long-term exposure. Indeed, LC-MUFA may even attenuate the cytotoxic actions of LC-SFA in rodent β-cells [3, 8, 9].

The mechanisms by which long-term LC-SFA treatment induce the demise of β-cells are unclear and the reasons that this response is attenuated by co-incubation with LC-MUFA are even less well established. Some insights into the underpinning mechanisms have been gained predominantly using multi-omics and molecular biology techniques (e.g. [8, 10–17]); and these have identified various intracellular signalling pathways which contribute towards β-cell lipotoxicity. However, the data are often conflicting, and few studies have revealed the mechanisms underpinning cryoprotection by unsaturated FFA. Potential pathways for cytotoxicity include a general perturbation of intracellular lipid homoeostasis [18], the accumulation of reactive oxygen species [19], increased endoplasmic reticulum (ER) stress [10] mitochondrial dysfunction [11] and altered rates of autophagy [12]. In addition, gross changes to particular subcellular compartments have also been noted in cells exposed to LC-SFA, including in the ER, mitochondria and lipid droplets [10, 11, 13, 14], suggesting that biochemical and cellular perturbations may converge at such locations. A feature of many of these mechanisms is that they are compartmentalised, often focussed at the level of individual subcellular organelles such as the ER, lipid droplets, mitochondria etc. Therefore in the present study, we have taken a broader approach by considering more widely, the intracellular distribution of LC-SFA and its associated alterations in subcellular morphology. We believe that this will assist in elucidating the extent to which proposed mechanisms may contribute to lipotoxicity.

Recent studies undertaken with the human EndoC-βH1 β-cell line [20] have revealed a marked resistance to the toxic effects of palmitate [21–23]. Therefore, we also considered it instructive to compare the intracellular disposition of the LC-SFA palmitate in EndoC-βH1 cells vs rodent INS-1 cells under conditions when differential effects on viability are observed. Overall, our goal was to gain a deeper understanding of the factors that determine the sensitivity of each of these cell types to the LC-SFA palmitate, the most abundant LC-SFA in vivo [24].

## Materials and methods

### Cell culture

Insulin secreting INS-1E and INS-1 823/13 cells were used in this study and cultured as described by Asfari et al. [25] and Hohmeir et al. [26], respectively. EndoC-βH1 cells were cultured as detailed by Ravassard et al. [20]. Cell lines were routinely tested for mycoplasma contamination. For individual experiments, cells were seeded in a 12 well plate at 0.5 × 10^6^ cells/well and incubated for 24 h in complete medium. For imaging studies, cells were grown on glass coverslips to a 70% confluency and for live cell imaging were expanded in 35 mm culture dishes (World Precision Instruments, Sarasota, FL, USA) to a confluency of 30%. After 24 h, the extracellular medium was removed and replaced with culture medium devoid of foetal bovine serum (FBS) or bovine serum albumin (BSA) but containing the appropriate fatty acid-BSA complexes.

### Fatty acid preparation and viability analysis

Fatty acid/BSA complexes were prepared as detailed in [8]. Cell death was estimated using vital dye staining (Trypan Blue 0.4% wt/vol in PBS; Merck, Darmstadt, Germany) as described previously by Welters et al. [3]. Experiments were repeated independently a minimum of three times.

### Immunofluorescence and confocal microscopy

A stock solution of BODIPY™ FL C_16_ (ThermoFisher, Gloucester, UK) was prepared in 100% ethanol and bound to BSA/fatty acid complexes to give a final concentration of 400 nM. After 24 h, cells were treated with the relevant BODIPY™ FL C_16_ /BSA/fatty acid complexes for the desired duration. Cells were fixed in 4% (w/v) paraformaldehyde and washed in phosphate buffered saline (PBS) prior to mounting. When probing for PLIN2, fixed cells were permeabilised with 0.2% Triton X-100 and incubated overnight with an anti-PLIN2 antibody (see supplementary file 1: Table 1 for details and conditions of the antibodies used). Cells were then incubated with species-specific fluorescently labelled secondary antisera and the cell nuclei stained with DAPI. Cells were then mounted. When BODIPY™ FL C_16_ localisation to the Golgi apparatus was examined, the Golgi apparatus was first stained with CellLight® Golgi-RFP (BacMam 2.0, ThermoFisher). CellLight® Golgi-RFP was prepared according to the manufacturer’s instructions and added to cells at 50 particles per cell (ppc) directly in culture medium 24 h after seeding. BODIPY FL C_16_ /BSA/fatty acid complexes were then added 24 h later. Cells were maintained at 37 °C during live cell imaging.

Images were captured with a DMI8 TCS SP8 confocal microscope (Leica Microsystems, Milton Keynes, UK). ImageJ/FIJI version 1.50b (https://imagej.nih.gov/ij/) was used for quantification studies. To determine the total area of the cell occupied by lipid droplets, a region of interest was selected and the total area of the cell covered by lipid droplets was divided by the total cell area and the results expressed as a percentage. The quantitative co-localisation analysis for Golgi-RFP, PLIN2 and BODIPY™ FL C_16_ was conducted using ImageJ/FIJI Coloc2 (https://imagej.net/Coloc_2), whereby a Pearson correlation coefficient was calculated to quantify the degree of co-localisation between fluorescent probes. Quantification studies were repeated for a minimum of five cells per condition.

### Electron microscopy

Monolayers of cells were fixed in 1% (w/v) glutaraldehyde and 2% (w/v) paraformaldehyde in 0.1 M sodium cacodylate buffer (pH 7.2) and stored at 4 °C. Cells were then processed and imaged as described previously [27] and imaged using a JEOL JEM 1400 transmission electron microscope operated at 120 kV. Images were taken with a digital camera (ES 1000 W CCD, Gatan, Abingdon, UK).

### Western blotting

Western blotting was performed as described previously [12]. The conditions and details of the antibodies are provided in supplementary file 1, Table 1. Bands were detected using enhanced chemiluminescence. Immunoblots were scanned using the Bio-Rad GS-800 calibrated densitometer. Blot oversaturation was excluded by analysis with Quantity-one (Bio-Rad) software and Fiji (ImageJ) software and blots deemed to be within the linear range were used for quantification.

### Data analysis

Results are expressed as mean + standard deviation (SD). Statistical analysis was carried out using GraphPad Prism version 8.0 (https://www.graphpad.com/scientific-software/prism/). Statistical significance between mean values was calculated by ANOVA (with post hoc Tukey’s test) and regarded as significant when *P* < 0.05.

## Results

### Effect of long-chain fatty acids on β-cell viability

**Fig. 1:**
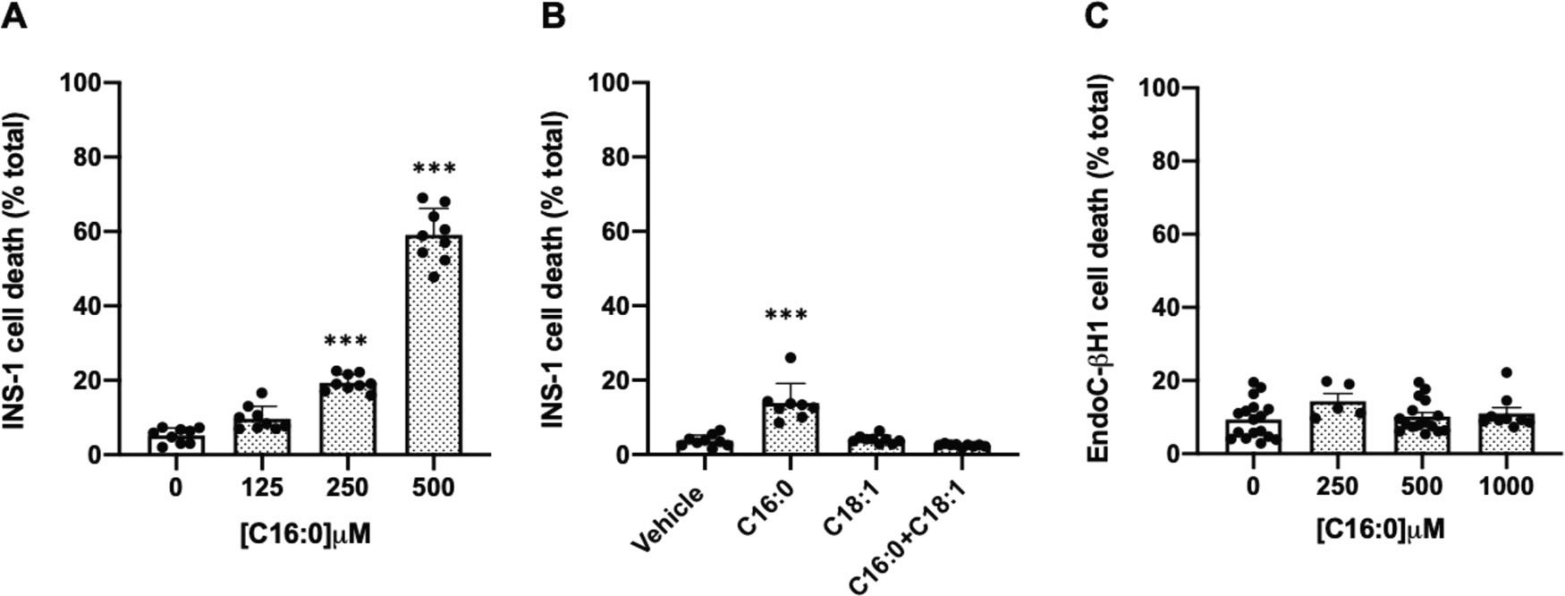
Effects of palmitate (C16:0) on the viability of rodent and human β-cells. Rat INS-1E (**A**) and human EndoC-βH1 (**C**) cells were treated with vehicle (0 µM), 125 µM, 250 µM, 500 µM or 1000 µM C16:0 for 24 h or 72 h, respectively. INS-1E (**B**) cells were treated with vehicle (0 µM) or 250 µM C16:0, either alone or in combination with C18:1 (250 µM) for 24 h. Cell death was estimated using vital dye staining. Dots represent individual data points and the histograms represent mean values + SD (*N* = 3). ***P < 0.001 relative to vehicle (0 µM).

Confirming previous studies [3, 8], palmitate exposure for 24 h caused a dose-dependent increase in INS-1E cell death (Fig. 1A) but this response was attenuated when the LC-MUFA, oleate, was also present in the incubation medium (Fig. 1B). By contrast, incubation with palmitate failed to induce EndoC-βH1 cell death, even at concentrations of up to 1 mM and for exposure periods of 72 h (Fig. 1C). Thus, the LC-SFA palmitate is well tolerated by human-derived EndoC-βH1 β-cells but is toxic to rat-derived INS-1E β-cells.

### Lipid droplet induction in INS-1E and EndoC-βH1 β-cells exposed to long-chain fatty acids

**Fig. 2:**
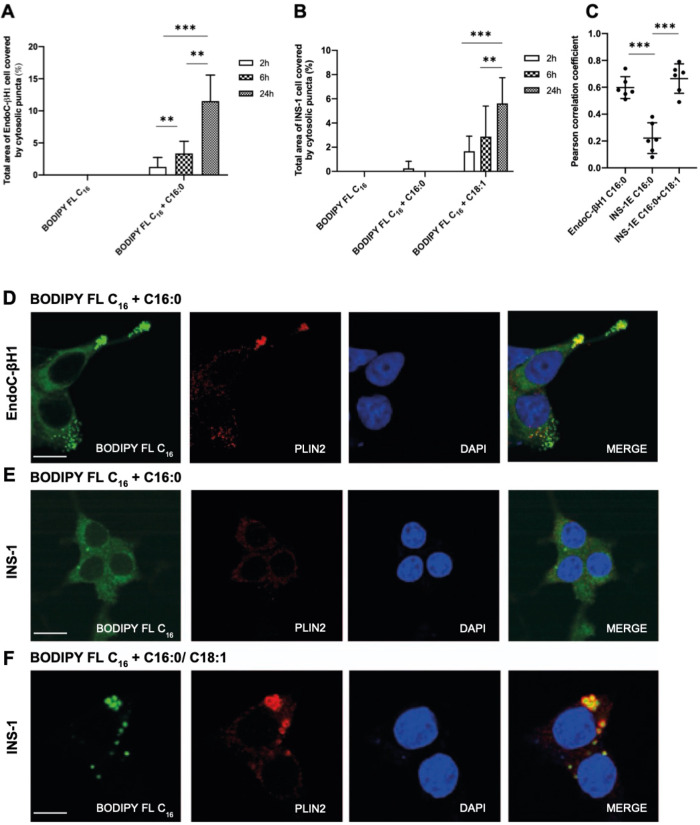
Cytoplasmic lipid droplets form rapidly in EndoC-βH1 exposed to palmitate (C16:0), but this does not occur in INS-1E cells unless oleate (C18:1) is also present. Quantification of the total area of EndoC-βH1 (**A**) and INS-1E (**B**) cells covered by fluorescent puncta after treatment with C16:0 (500 μM) and BODIPY FL C_16_ (400 nM) alone or in INS-1E cells, in combination with 250 μM C18:1 for 2 h, 6 h or 24 h. Images were analysed using ImageJ/FIJI software where the area of cell covered by fluorescent puncta was divided by the total cell area, and the results expressed as a percentage of the total. **C** Pearson correlation coefficient demonstrating the co-localisation BODIPY FL C_16_ with PLIN2. Dots represent individual data points. Representative immunofluorescence staining of BODIPY FL C_16_ (green), PLIN2 (red), and DAPI (blue) in EndoC-βH1 (**D**) and INS-1E (**E**, **F)** cells following a 24 h treatment with C16:0 [500 μM] and BODIPY FL C_16_ [400 nM] alone or in INS-1E cells, in combination with 250 μM C18:1 for 24 h. Merged images included to show co-localisation. Scale bars 10 μm. Data represents mean + SD (*N* = 3). **p < 0.01, ***p < 0.001 as indicated.

Next, the fluorescent palmitate analogue, BODIPY FL C_16_, was employed as a tracer to study the subcellular distribution of palmitate in both INS-1E and EndoC-βH1 cells. Incubation of EndoC-βH1 cells with BODIPY FL C_16_ in the presence of exogenous palmitate led to the appearance of fluorescent signal throughout the cytosol within 2 h. The fluorescence was markedly punctate in appearance and the total area of the cell covered by fluorescent puncta increased fourfold over 24 h (Fig. 2A, Supplementary File 1: S1a). Unlike the situation seen in EndoC-βH1 cells, the BODIPY FL C_16_ tracer did not accumulate in the cytoplasm of INS-1E cells suggesting a difference in the way palmitate is handled by the two cell types. Importantly, when oleate was also included in the incubation medium together with palmitate and the fluorescent tracer (Fig. 2B, Supplementary File 1: S1b, c) cytoplasmic puncta were then seen in INS-1E cells. Under these conditions, a threefold increase in the total area of the cell covered by fluorescent puncta occurred over a 24 h time course. To establish whether the cytosolic puncta might correspond to lipid droplets (LD), confocal co-immunofluorescence studies were performed to monitor the extent of colocalisation between BODIPY FL C_16_ and the protein, perilipin 2 (PLIN2) a component of LDs. This revealed a very clear co-localisation of the fluorescent signal arising from BODIPY FL C_16_ with the immunostaining of PLIN2 in EndoC-βH1 cells exposed to palmitate (Fig. 2C, D). Similar findings were made in INS-1E (Fig. 2C, F) cells co-treated with both palmitate and oleate, but no such co-localisation occurred in INS-1E cells treated with palmitate alone (Fig. 2C, E). Notably, when treated with BODIPY tracer only (in the absence of additional exogenous palmitate), no LD were observed in either cell line (Supplementary File 1: Fig. S2), indicating that the cytosolic distribution of BODIPY FL C_16_ was dictated by the disposition of the exogenous fatty acid rather than by the fluorophore itself.

### Electron microscopy analysis of subcellular organelle morphology in β-cells following treatment with long-chain fatty acids

**Fig. 3:**
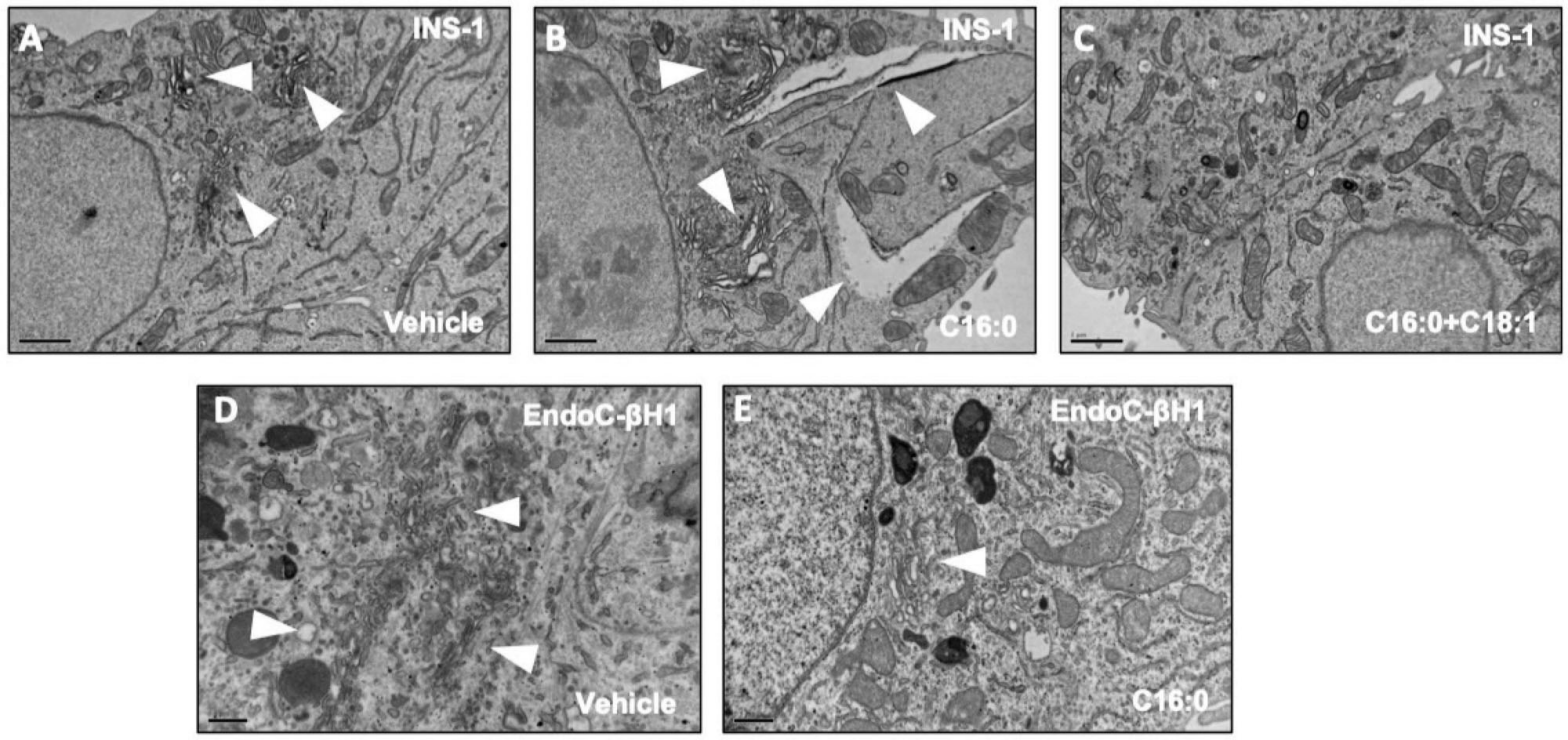
Effect of palmitate (C16:0) on the morphology of rodent and human β-cells. Representative transmission electron microscopy images of INS-1 823/13 cells (**A**–**C**) and EndoC-βH1 (**D**, **E**) cell morphology following a 6 h treatment with BSA vehicle control (**A**, **D** ‘vehicle’) or 250 μM C16:0 (**B**, **E**) without or with 250 μM C18:1 (**C**) (*N* = 2). Arrows point towards the Golgi apparatus and the distension of intracellular membranes.

We next determined the changes occurring at the subcellular level in EndoC-βH1 and INS-1 821/13 cells treated with LC-FFAs, using electron microscopy (EM). Examination of the EM images revealed important morphological differences between INS-1 and EndoC-βH1 cells following exposure to palmitate. The intracellular membranes of INS-1 cell organelles were distended when compared with control cells (Fig. 3A and B; Supplementary file 1: S3) and the Golgi apparatus swollen (Fig. 3B; supplementary file 1: S4). Such changes were not observed in control cells treated with vehicle (Fig. 3A) or when the cells were co-treated with palmitate and oleate together (Fig. 3C). No observable changes in the Golgi apparatus morphology (Fig. 3E) or in the morphology of other intracellular membranes (Fig. 3D and E) were visible in EndoC-βH1 cells following treatment with palmitate, by comparison with cells exposed to vehicle only.

**Fig. 4:**
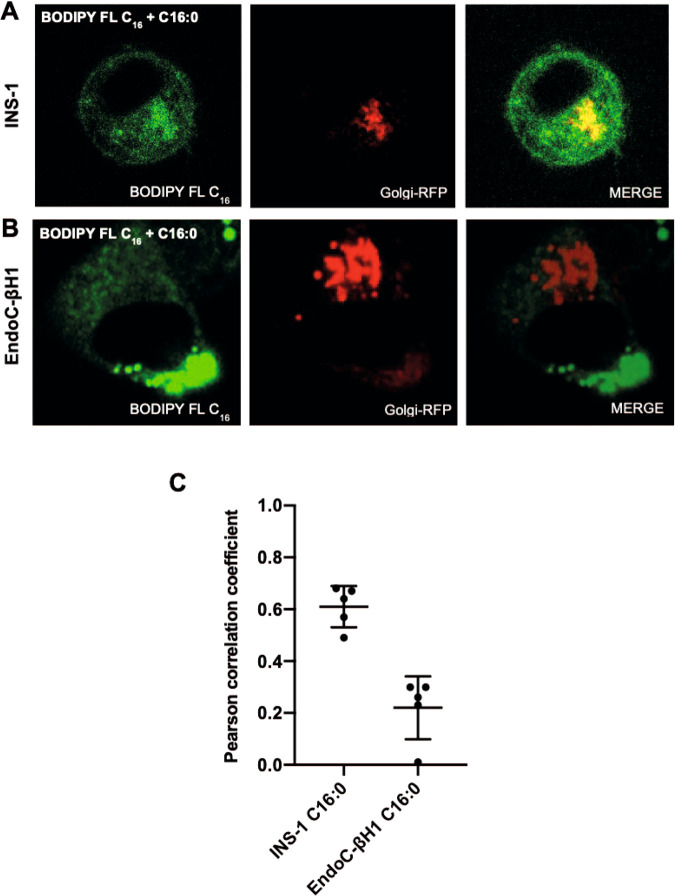
Palmitate (C16:0) accumulates in the Golgi apparatus of rodent but not human β-cells. The Golgi apparatus of INS-1E (**A**) and EndoC-βH1 (**B**) cells was stained with CellLight Golgi-RFP (red) and exposed to BODIPY FL C_16_ (green) with 250 μM C16:0 for 24 h. Merged images included to show co-localisation. **C** Pearson correlation coefficient demonstrating the co-localisation of BODIPY FL C_16_ and CellLight Golgi-RFP. Dots represent individual data points. Data are mean + SD (*N* = 3).

In view of the observation that palmitate induces swelling of the Golgi apparatus in INS-1 (but not EndoC-βH1) cells, the Golgi apparatus of both EndoC-βH1 and INS-1E cells was labelled with the red fluorescent marker, CellLight® Golgi-RFP in the presence of BODIPY FL C_16_ and exogenous palmitate. Surprisingly, BODIPY FL C_16_ was seen to co-localise with the Golgi marker very rapidly in INS-1E cells (Fig. 4A, C; Supplementary File 1: S5) but this did not occur in EndoC-βH1 cells (Fig. 4B, C).

### ER-stress induction in rat INS-1E and human EndoC-βH1 cells exposed to long-chain fatty acids

**Fig. 5:**
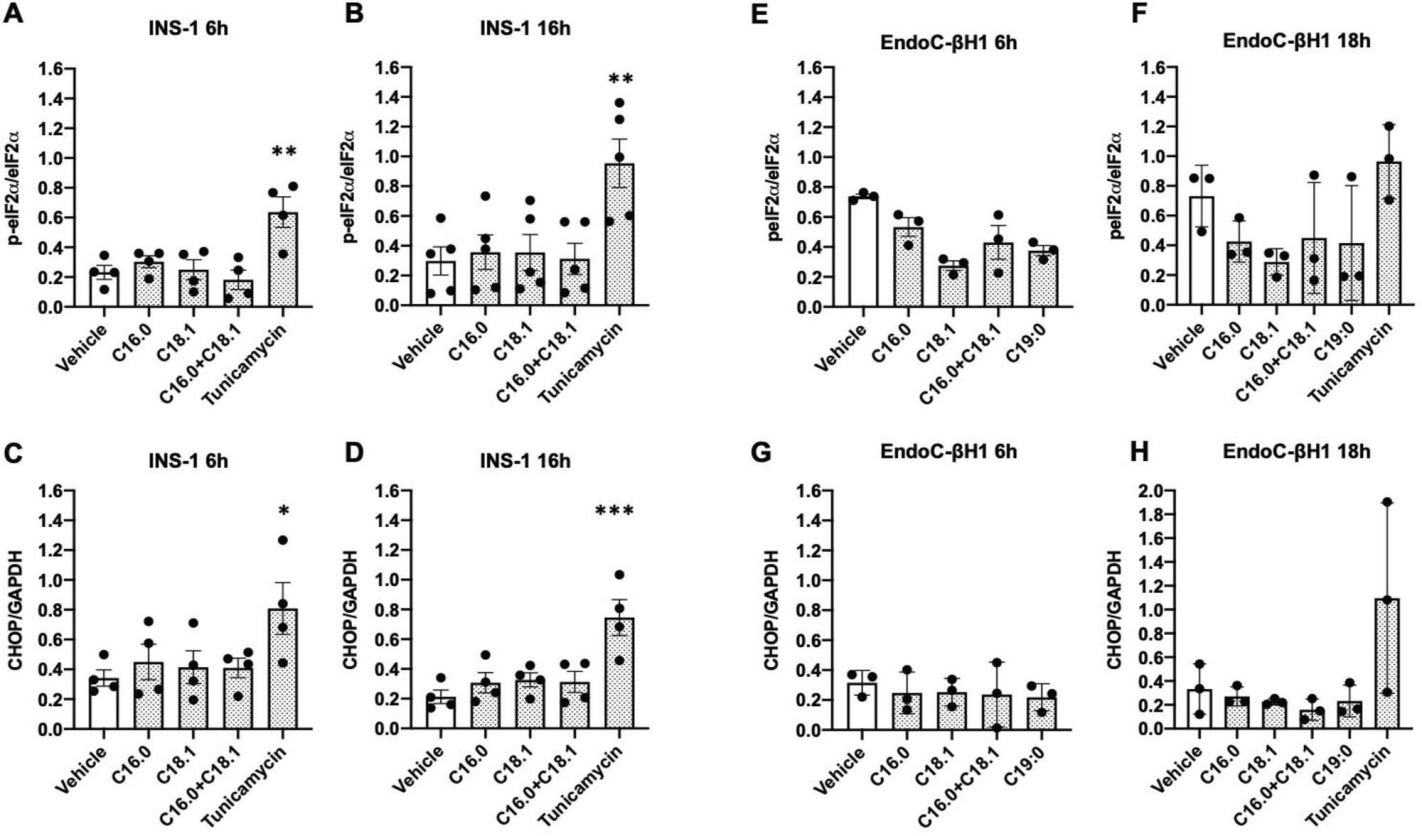
LC-FFA do not activate ER stress in rodent or human β-cells. INS-1E (**A**–**D**) and EndoC-βH1 cells (**E**–**H**) were treated with either 250 μM C16:0, 250 μM C18:1, 250 μM C19:0, 250 μM C16:0 + 250 μM C18:1, or 5 μg/ml Tunicamycin for 6 h,16 h or 18 h. Total eIF2a, peIF2a, CHOP and GAPDH levels were analysed by Western blotting and quantified as peIF2α/total eIF2α ratio or CHOP/GAPDH ratio. Dots represent individual data points. Data are mean + SD (*N* = ≥ 3). * *P* < 0.05, **P < 0.01, ***p < 0.005 relative to control.

Previously, we have shown that palmitate-induced intracellular membrane distension correlates with activation of the PERK-dependent arm of the ER stress pathway in rat BRIN-BD11 cells [8]. In the present study, however, immunoblotting revealed that, under the conditions employed, palmitate did not promote activation of the PERK-dependent ER stress pathway in INS-1E cells (Fig. 5A, D, Supplementary File 1: S6). Thus, exposing INS-1E cells for either 6 h or 16 h with palmitate or oleate alone, or with palmitate plus oleate together, failed to induce the phosphorylation of eIF2α (Fig.  5A, B), or the expression of CHOP-10 (Fig. 5C, D), both of which are typically associated with activation of the PERK pathway of ER stress. As expected, tunicamycin, a known inducer of PERK-mediated ER stress, promoted eIF2α phosphorylation and a significant rise in CHOP-10 expression in INS-1E cells.

As seen in INS-1E cells, palmitate was also ineffective as an activator of the PERK-dependent ER stress pathway in EndoC-βH1 cells (Fig. 5E–H, Supplementary File 1: S7). Surprisingly, tunicamycin also failed to significantly induce eIF2α phosphorylation and CHOP-10 upregulation in these cells following 18 h of culture.

## Discussion

We have shown that rat INS-1 β-cells rapidly incorporate exogenous palmitate into the membranes of intracellular organelles, including the Golgi apparatus. This resulted in marked changes to their intracellular morphology when viewed under the electron microscope, consistent with earlier studies in BRIN-BD11 β-cells [8] and in other, non-endocrine, cell types [28]. Contrary to studies using the INS-1 cell line [10, 14, 16], these changes were not associated with induction of the PERK-dependent arm of the ER stress pathway in INS-1E cells. This observation suggests that the morphological and biochemical changes occurring when cells are exposed to palmitate do not necessarily lead to the activation of stress responses focussed in the ER.

Consistent with a failure to induce ER stress, we found that the fluorescent analogue of palmitate accumulated very rapidly in the Golgi apparatus of INS-1E cells, suggesting that it was not concentrated primarily within the ER. Rather, it appears that palmitate is rapidly routed to the Golgi apparatus in INS-1 cells, thereby implying that traffic from the ER to the Golgi can proceed efficiently, despite the presence of the fatty acid. This might then account for the failure of palmitate to promote ER stress in INS-1E cells, under the conditions of our experiments.

Irrespective of the early disposition of palmitate, incubation of INS-1E cells with this fatty acid led to a loss of viability. Thus, even though palmitate failed to promote an ER stress response (or, perhaps, because of this), the cells were unable to maintain their viability when exposed to this fatty acid alone, and the majority succumbed within 18–24 h. This stands in marked contrast to the outcome when cells were exposed to palmitate together with oleate. Under these conditions, viability was maintained despite the presence of total exogenous fatty acid concentrations as high as 0.5 mM.

Importantly, the disposition of palmitate (as judged using the fluorescent tracer BODIPY FL C_16_) was very different in INS-1E cells exposed simultaneously to oleate by comparison with those treated with palmitate alone. When both fatty acids were present together, no accumulation of the tracer was seen in the Golgi apparatus but, rather, it was concentrated within LD. For human EndoC-βH1 cells, exogenous palmitate was also routed into LD, although, unlike the situation in INS-1E cells, this occurred in the absence of oleate. In EndoC-βH1 cells, palmitate did not accumulate in the Golgi apparatus under any conditions studied and, as in INS-1 cells, palmitate did not elicit a PERK-dependent ER stress response. Moreover, we found that the ER stress-inducing antibiotic, tunicamycin, failed to induce ER stress in EndoC-βH1 cells, supporting the work of Oleson et al. [29] who demonstrated that ER stress was not induced when EndoC-βH1 cells treated with the Ca^2+^ ATPase (SERCA) inhibitor, thapsigargin.

Taken together, our findings suggest that there are marked differences in the handling of the LC-SFA palmitate, when this FA is added exogenously to rodent or human β-cells. This supports the proposition that lipotoxicity may be a phenomenon occurring primarily in rodent β-cells, as posed by Weir [4]. Oshima et al. [21] have proposed that EndoC-βH1 cells may be resistant to the toxic effects of palmitate due to the abundant expression of a FA desaturase enzyme, stearoyl CoA desaturase (SCD), which converts LC-SFA to their monounsaturated counterparts. Since there is evidence that LC-SFA can promote cell death in isolated primary human islets [9, 10] future research should aim to determine whether the response to palmitate in human EndoC-βH1 cells is a feature of the cell line or a true reflection of the situation in vivo.

We have found that the viability of INS-1E and EndoC-βH1 cells is maintained when exogenous LC-FFA are routed towards cytoplasmic LD formation, suggesting that trafficking of LC-SFA into LD may confer protection against lipotoxicity in β-cells. In agreement with our results, previous studies [13, 30] have reported a time-dependent formation of LD in rodent β-cells co-treated with palmitate and oleate. Furthermore, others have observed LD in the cytoplasm of EndoC-βH1/2 cells following prolonged exposure to palmitate [30, 31]. Interestingly, the overexpression of LD-associated proteins, PLIN1 and PLIN5, has been shown to protect rodent β-cell function and viability during exposure to exogenous palmitate via an increase in FFA oxidation and a decrease in ER stress [15, 32]. However, silencing of PLIN1 and PLIN2 to reduce LD formation did not potentiate palmitate-induced rodent β-cell death when cells were co-treated with oleate [30]. Therefore, the role of LD in the protection of β-cells against lipotoxicity merits further study.

To the best of our knowledge, we are the first to report that palmitate accumulates in the Golgi apparatus of INS-1 β-cells at early time points. In drawing this conclusion, we note however, that, Karaskov et al. [14] also observed morphological changes to the Golgi in rodent β-cells following an acute treatment with palmitate. Emerging research has shown that morphological and functional changes to the Golgi can trigger apoptosis, which may then contribute to the development of a range of diseases including cardiovascular, neurodegenerative diseases and cancer (as discussed in Liu et al. [33]). Furthermore, the Golgi can transmit and receive a range of signals that have been shown to influence other intracellular processes including apoptosis [34], stress responses [35], autophagy [36], and the prevention of the toxic accumulation of ceramides [37]. These findings concur with the proposed mechanisms underpinning β-cell lipotoxicity [10, 12, 18, 38]. As a result, we believe that changes to the morphology and function of the Golgi apparatus in β-cell lipotoxicity require additional investigation to determine the role it played in causing cell death.

We have previously reported that distension of the ER membrane in rodent BRIN-BD11 β-cells is associated with the activation of ER stress in palmitate-treated cells [8]. We were therefore surprised to find that palmitate did not activate the ER stress response in INS-1E cells. Bachar et al. [16]. reported only a modest increase in CHOP and eIF2α phosphorylation in palmitate-treated INS-1E cells unless also cultured in a high concentration of glucose. Marchetti et al. [17]. also found only modest signs of ER stress in isolated islets from T2D subjects, with ER stress being exacerbated upon culturing the islets in high concentrations of glucose. Collectively, these results suggest that enhanced ER stress is not the primary mechanism underlying β-cell lipotoxicity. We accept, however, that it cannot be ruled out that the reason for the disparity between our findings and those of others may be due to subtle differences in methodologies e.g. fatty acid composition and incubation times.

In summary, the present findings support the hypothesis that when exogenous palmitate is incorporated preferentially into intracellular membranes, pancreatic β-cell viability is compromised. By contrast, when palmitate is routed into LD, β-cell viability is maintained. Future work should study changes in the lipid composition of intracellular membranes and which intracellular organelles are affected, following a prolonged treatment with palmitate and establish the factors that determine the routing of this fatty acid within cells when it is supplied exogenously.

## Supplementary information

Supplementary file 1

Supplementary file 2

Video summary

## Data Availability

Data generated and analysed during this study are included within the published article (supplementary information file 2).
