# Supplementary material for: Differential routing and disposition of the long-chain saturated fatty acid palmitate in rodent vs human beta-cells
Source: Nutr Diabetes. 2022 Apr 20;12:22. doi: 10.1038/s41387-022-00199-y (PMC9021209; doi:10.1038/s41387-022-00199-y)
Supplement: Supplementary file 1 [file 41387_2022_199_MOESM1_ESM.pdf]

Table S1: Antibodies used within this study		
**Primary Antibody**	**Manufacturer, host and clonality**	**Conditions and secondary detection system**
**PLIN2**	Progen GP40<br>Guinea pig polyclonal	Immunofluorescent staining (1:100 for 16h at 4°C) using Fluor-conjugated goat anti-guinea pig secondary antibody (1:400 for 1h; Invitrogen)
**peIF2α**	Cell Signalling Technologies 3398P<br>Rabbit monoclonal	Western blotting (1:1000 for 16h at 4°C) using HRP-linked anti-rabbit secondary antibody (1:5000 for 1h; Agilent Dako)
**Total eIF2α**	Cell Signalling Technologies 5324P<br>Rabbit monoclonal	Western blotting (1:1000 for 16h at 4°C) using HRP-linked anti-rabbit secondary antibody (1:5000 for 1h; Agilent Dako)
**CHOP (INS-1)**	Santa Cruz sc-575<br>Rabbit polyclonal	Western blotting (1:200 for 16h at 4°C) using HRP-linked anti-rabbit secondary antibody (1:5000 for 1h; Agilent Dako)
**CHOP (EndoC-βH1)**	Proteintech Europe 15204-1-AP<br>Rabbit Polyclonal	Western blotting (1:500 for 16h at 4°C) using HRP-linked anti-rabbit secondary antibody (1:5000 for 1h; Agilent Dako)
**GAPDH**	Hytest 5G4<br>Mouse monoclonal	Western blotting (1:10,000 for 16h at 4°C) using HRP-linked anti-mouse secondary antibody (1:5000 for 1h; Thermo Fisher Scientific)

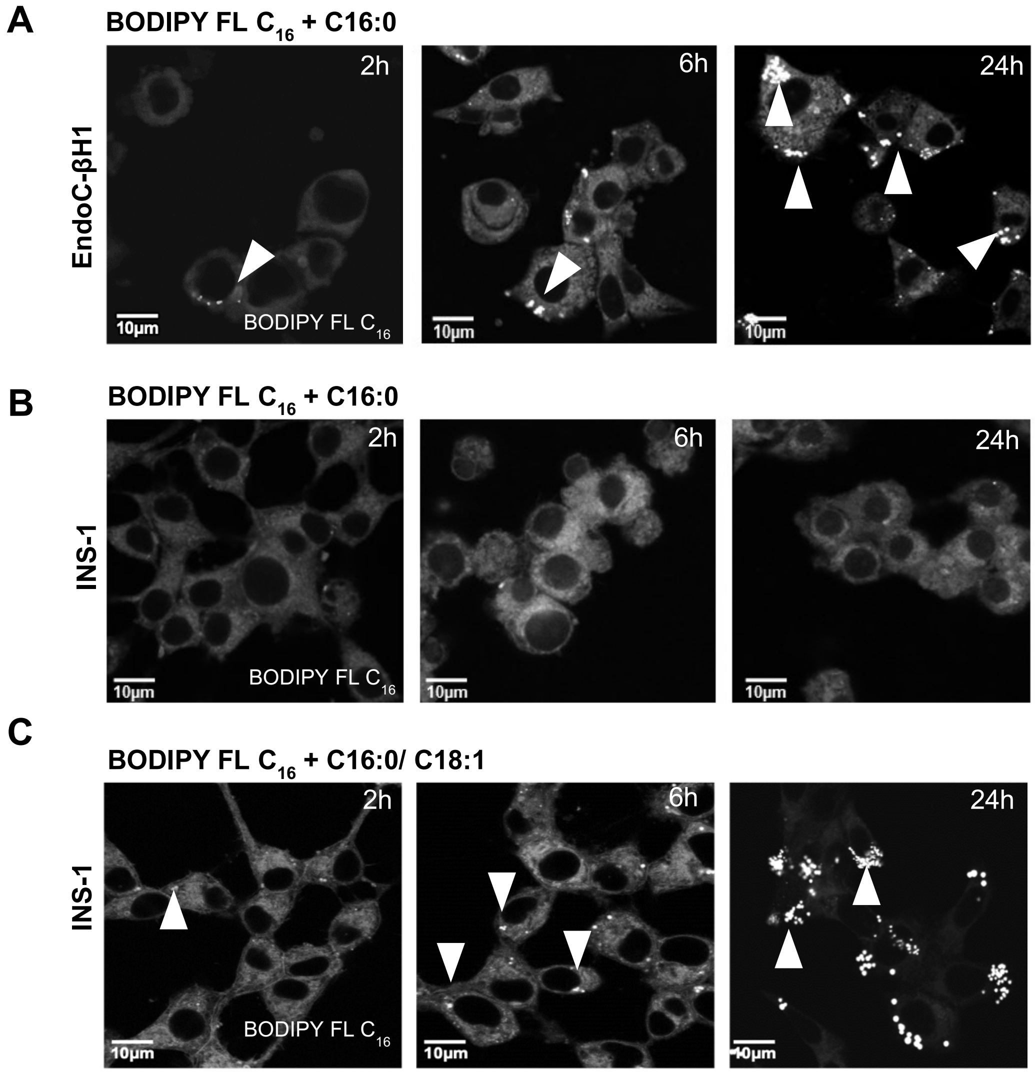

S2: Representative images of EndoC-βH1 **(A)** and INS-1 cells **(B)** exposed to C16:0 [500µM] BODIPY FL C_{16} [400nM] alone or in combination with 250µM C18:1 for 2h, 6h or 24h. Scale bars 10µm.

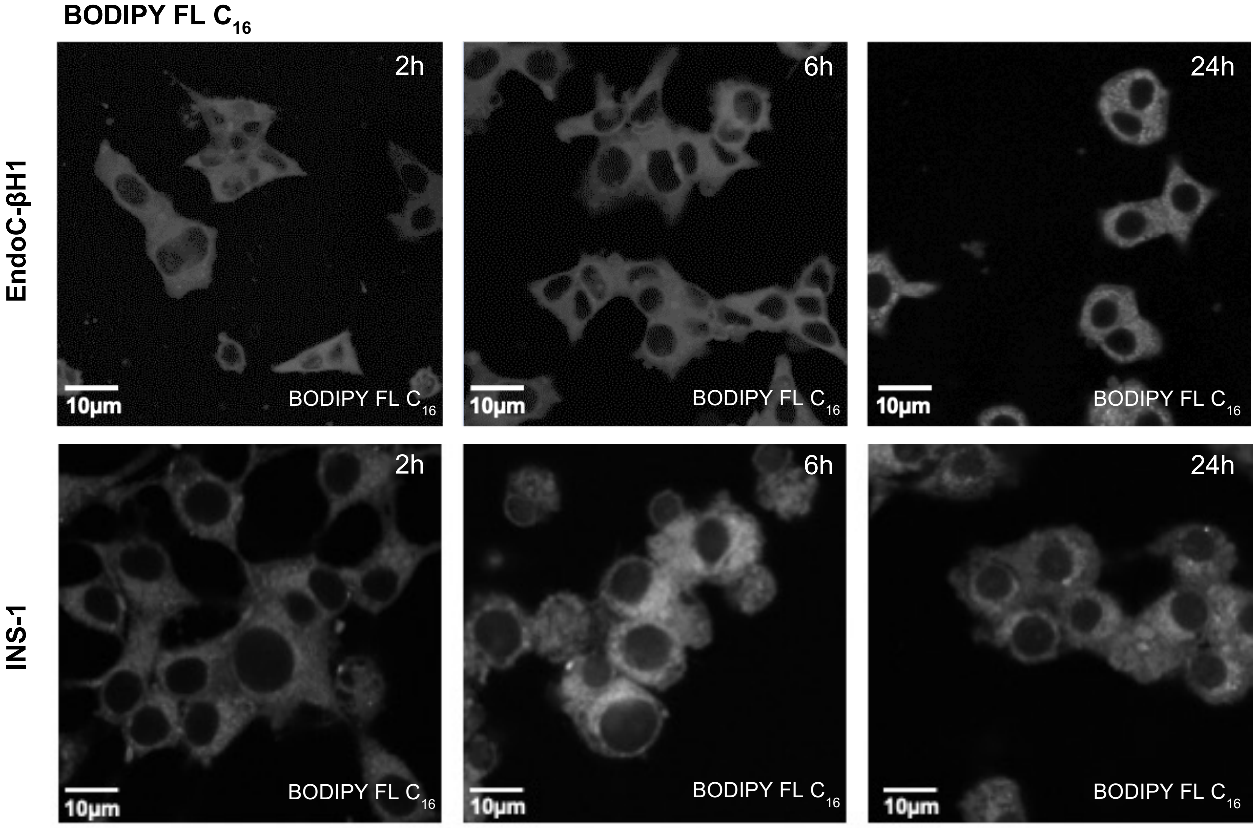

S3: Representative images of EndoC-βH1 **(A)** and INS-1 cells **(B)** exposed to the BODIPY FL C_{16} [400nM] tracer only for 2h, 6h or 24h. Scale bars 10µm.

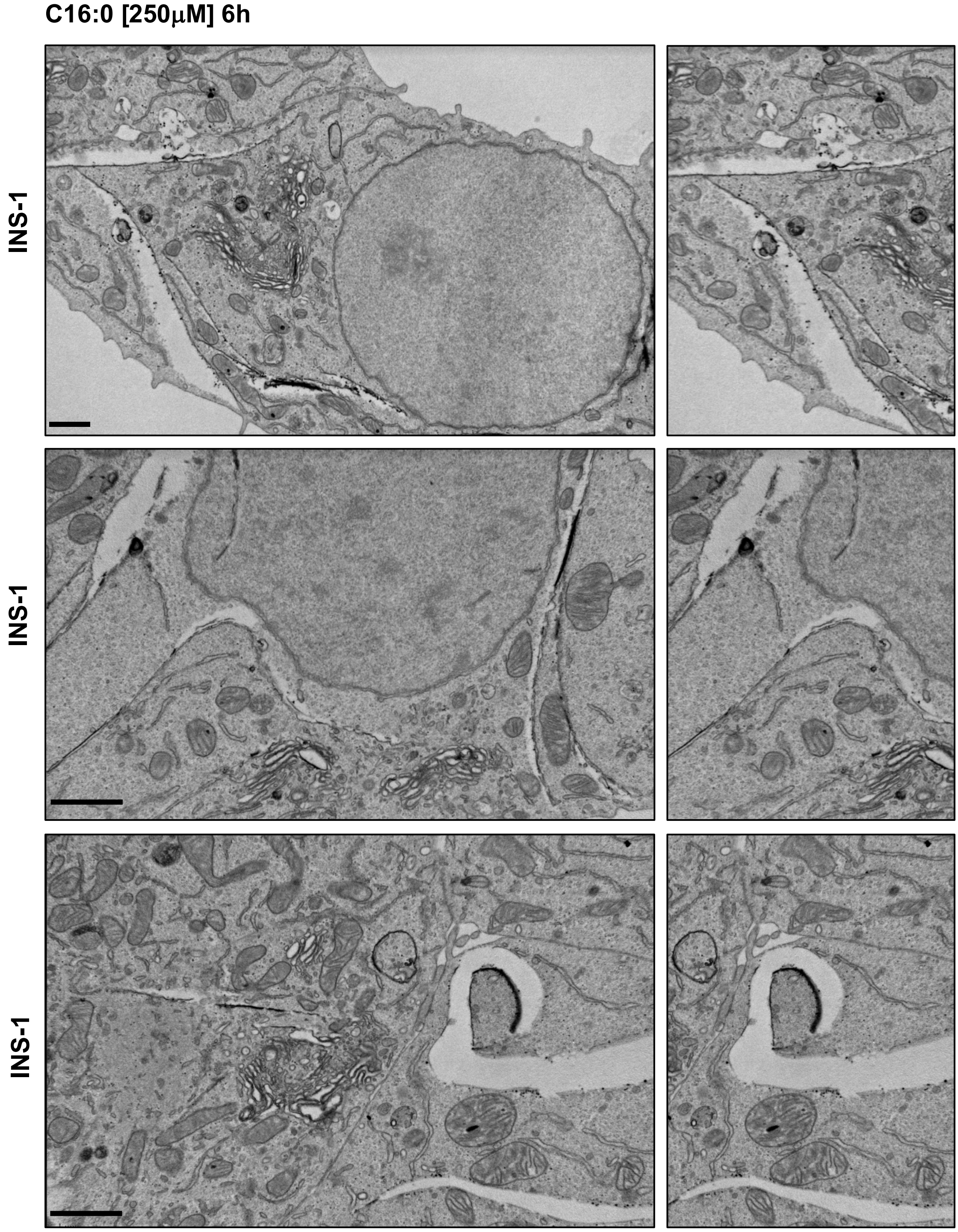

S4: The effect of C16:0 on intracellular membrane morphology in INS-1 cells**.** INS-1 cells were treated for 6hrs with 250μM C16:0. INS-1 cells were then fixed in an osmium tetroxide fixative before being imaged with TEM. Scale bar represents 1μm.

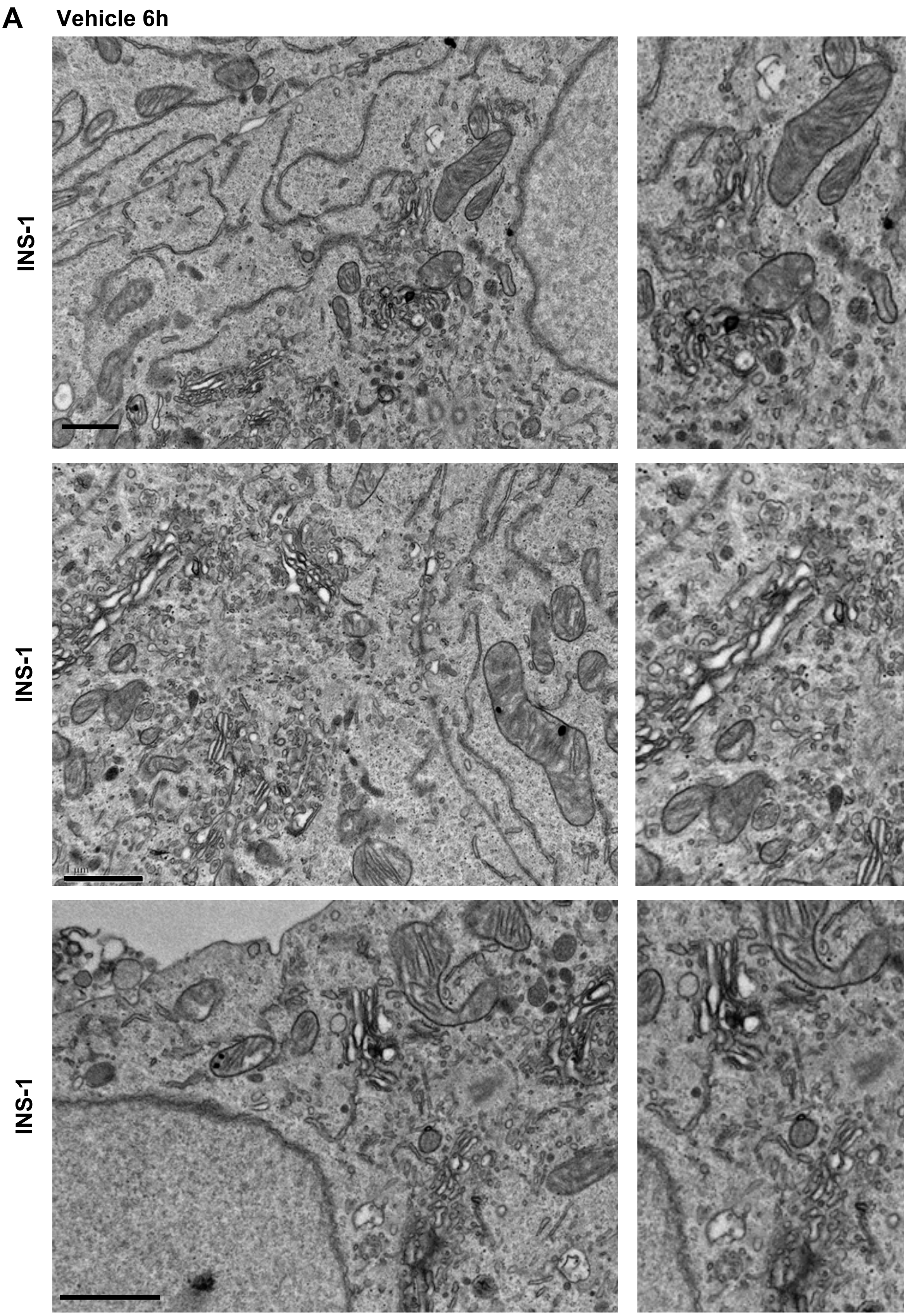
A
Vehicle 6h
INS-1
INS-1
INS-1

B **C16:0 [250μM] 6h**

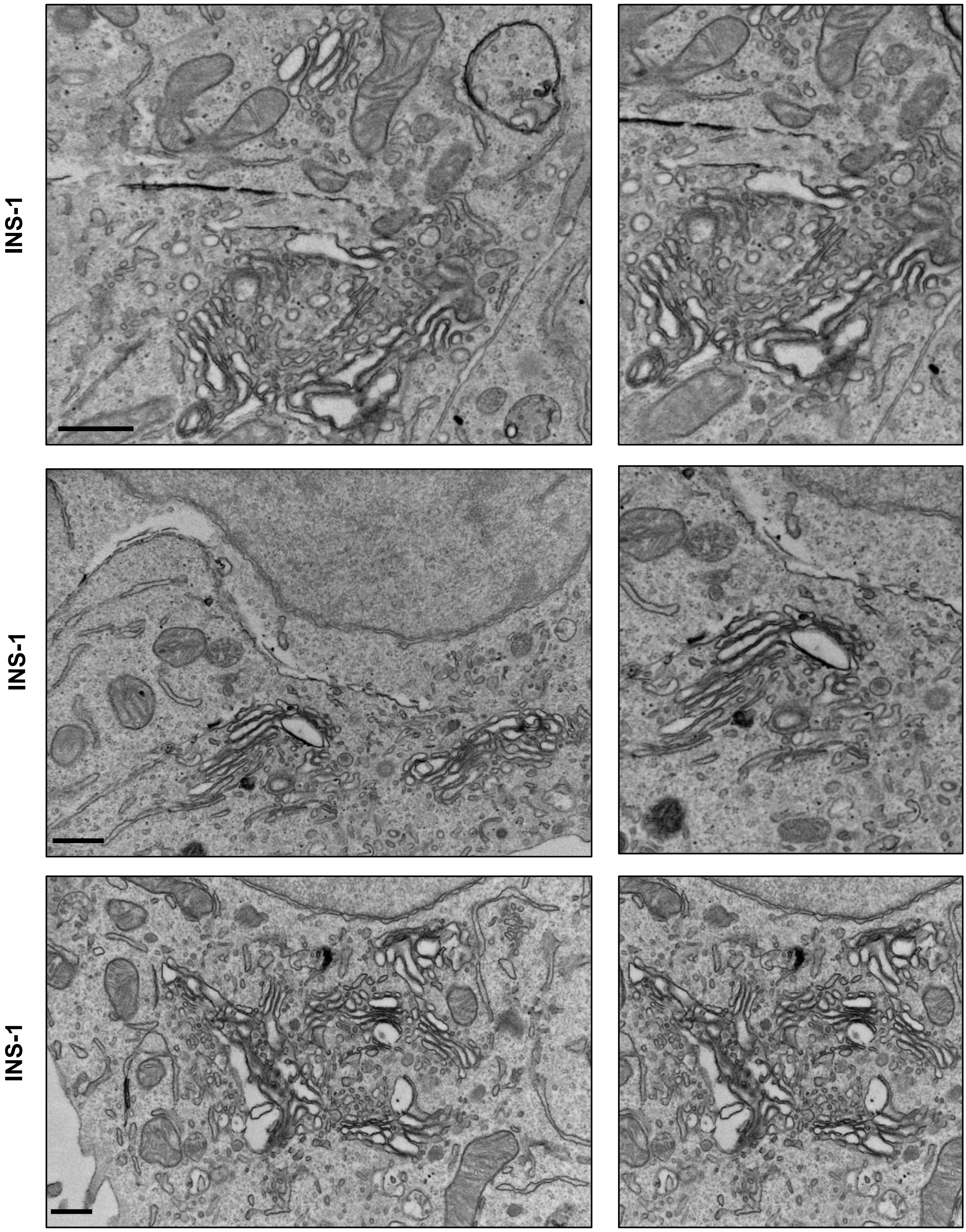

S5: The effect of C16:0 on Golgi morphology in INS-1 cells. INS-1 cells were treated for 6hrs with either vehicle control (**A**) or 250μM C16:0 (**B**). INS-1 cells were then fixed in an osmium tetroxide fixative before being imaged with TEM. Scale bar represents 1μm.

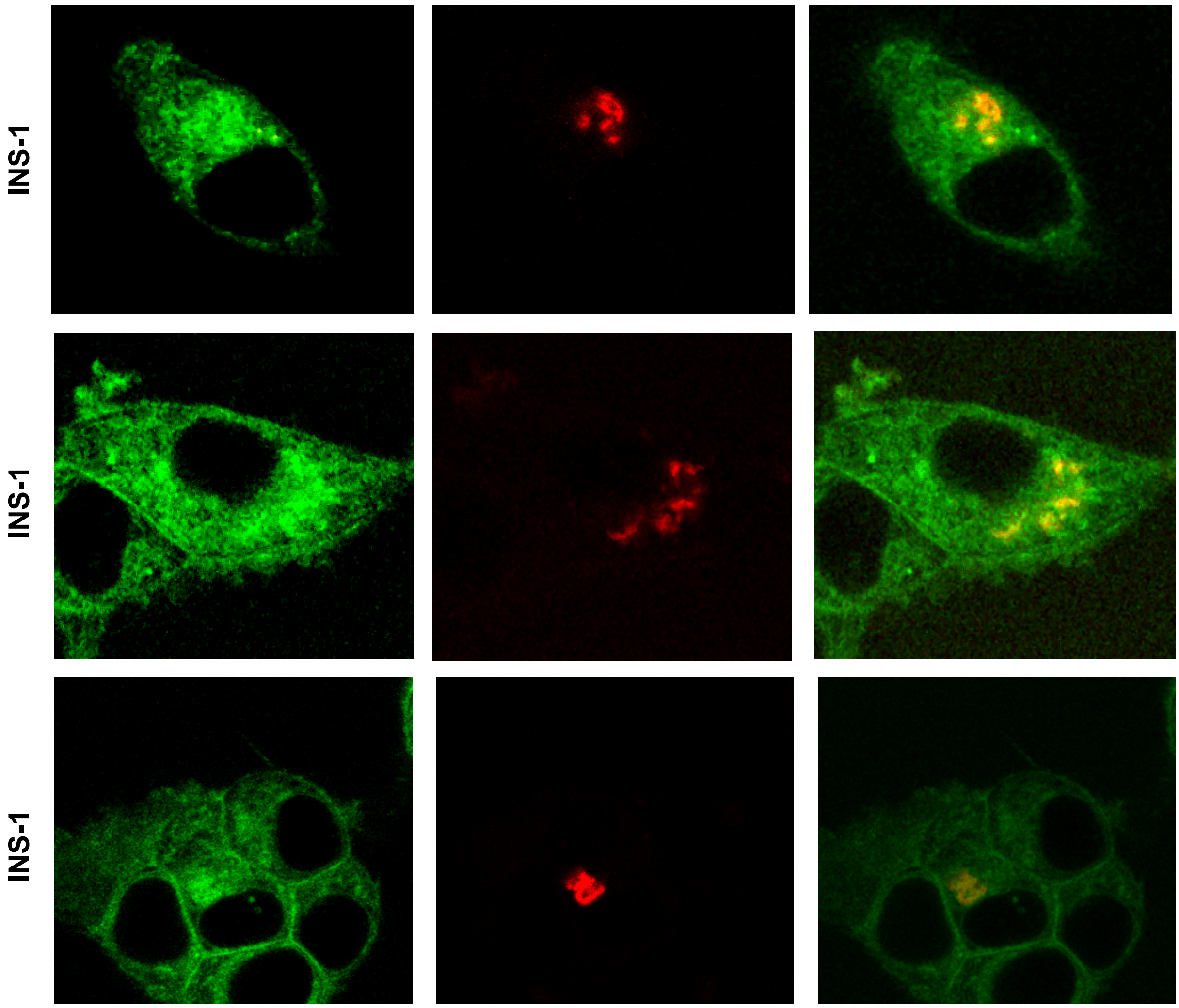

S6: Confocal fluorescent images of INS-1 cells where the Golgi apparatus was stained with CellLight Golgi-RFP and the cells exposed to BODIPY FL C_{16} with 250µM C16:0 for 24h.

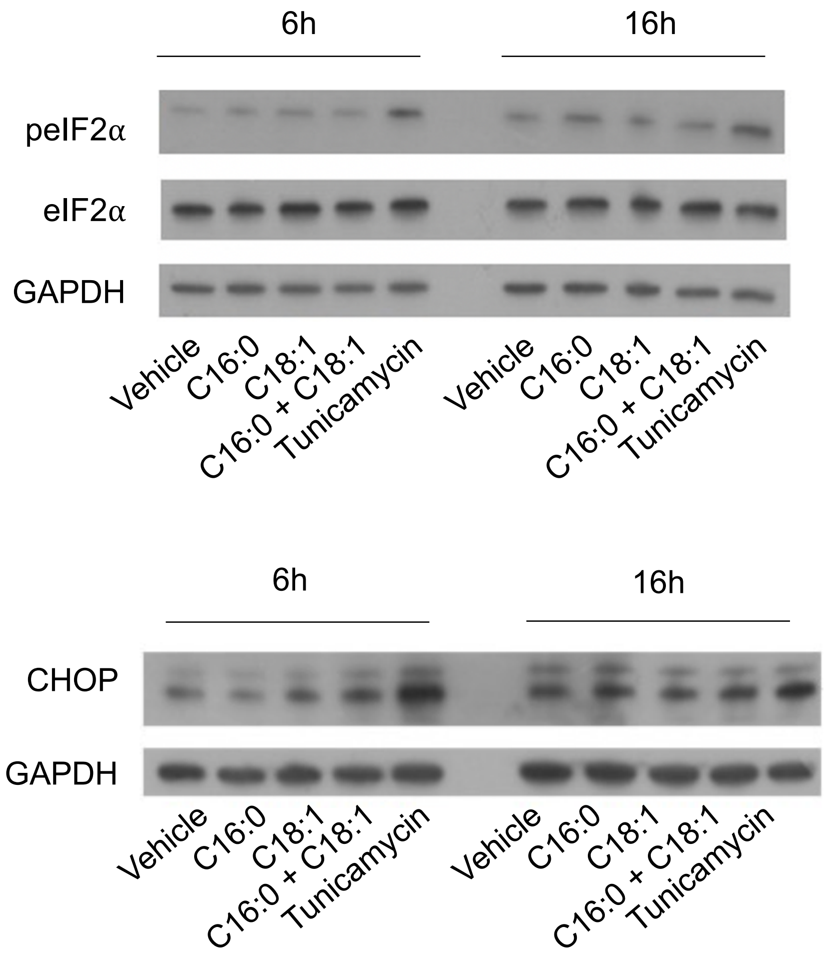

S7: Sample blots to show that LC-FFA do not activate ER stress in rodent INS-1 cells. INS-1 cells were treated with either 250µM C16:0, 250µM C18:1, 250µM 16:0 + 250µM C18:1, or 5µg/ml Tunicamycin for 6h or 16h. Total eIF2α, peIF2α, CHOP and GAPDH levels were analysed by Western blotting.

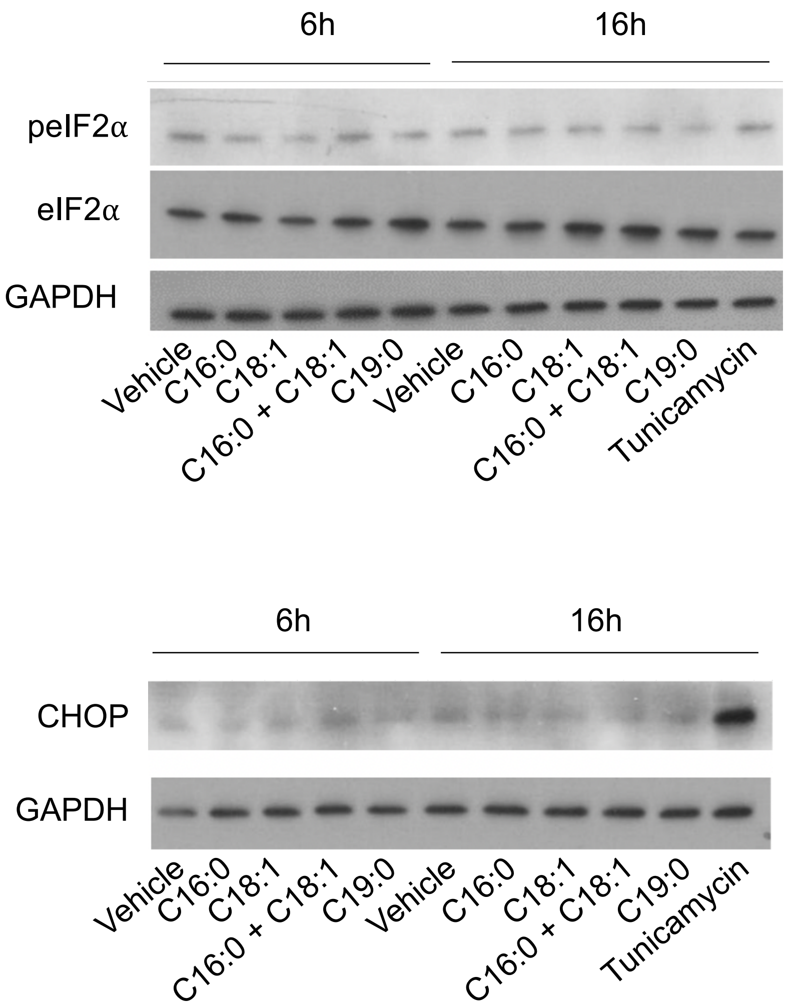

S8: Sample blots to show that LC-FFA do not activate ER stress in human EndoC-βH1 cells. EndoC-βH1 cells were treated with either 250µM C16:0, 250µM C18:1, 250µM C19:0, 250µM C16:0 + 250µM C18:1, or 5 µg/ml Tunicamycin for 6h or 18h. Total eIF2α, peIF2α, CHOP and GAPDH levels were analysed by Western blotting.
