# Supplementary material for: Differential routing and disposition of the long-chain saturated fatty acid palmitate in rodent vs human beta-cells
Source: Nutr Diabetes. 2022 Apr 20;12:22. doi: 10.1038/s41387-022-00199-y (PMC9021209; doi:10.1038/s41387-022-00199-y)
Supplement: Supplementary file 2 [file 41387_2022_199_MOESM2_ESM.pdf]

Raw data for Fig. 1: Effects of palmitate (C16:0) on the viability of immortal rodent-derived INS-1 and human-derived EndoC-βH1 β-cells.

Fig. 1A. INS-1 C16:0

Concentration	0	125	250	500
	3	13	19	64
	2	7	16	68
	3	10	18	69
	6.8	7	22.5	57
	4.8	7.7	22.2	47.8
	7.4	16.6	18.6	60.5
	7.3	10.6	17.1	52.3
	5.9	8.4	21.6	54.4
	6.4	7.3	19.1	58.9

Fig. 1B. INS-1 C16:0 + C18:1

Concentration	Vehicle	C16:0	C18:1	C16:0+C18:1
	1.5	14.3	2.8	2.7
	3.8	26	4.1	2.2
	3.2	13.4	3.6	3
	2.5	12.4	6.4	2.5
	4.2	13.7	3.7	2.6
	2.5	12.59	4.1	2.6
	6.53	8.5	4.17	2.22
	5.49	10.06	2.8	1.75
	3.48		4.77	2.66

Fig.1C. EndoC-βH1 C16:0

Concentration	0	250	500	1000
	19.5	12.4	11.9	9.5
	12.7	11.1	9.6	7.3
	10.3	9.7	19.5	10.2
	16.4	19	10.4	9.3
	18.1	19.8	8	8.6
	9.4		7.4	8.9
	6.2		6.4	22.2
	2.9		5.4	14.5
	5.7		6.1	9.1
	4		6.1	
	3.9		8.6	
	5.9		7.6	
	4.7		7.9	
	4.2		17.7	
	12.2		14.6	
	11		15.9	
	11.7			

Raw data for Fig. 2A: Quantification of the total are of EndoC-βH1 cells covered by fluorescent puncta after treatment with C16:0 (500µM) and BODIPY FL C16 (400nM).

A. EndoC-βH1 C16:0 lipid droplet (% total area of the cell covered by lipid droplet)

2h	BODIPY FL C_{16}	BODIPY FL C_{16} + C16:0	BODIPY FL C_{16} + C18:1
	0.00	0.77	0.90
	0.00	0.00	0.00
	0.00	1.78	3.08
	0.00	0.00	2.91
	0.00	0.00	2.47
	0.00	0.00	2.37
	0.00	0.00	1.64
	0.00	0.00	0.00
	0.00	0.00	
	0.00	0.00	

6h	BODIPY FL C_{16}	BODIPY FL C_{16} + C16:0	BODIPY FL C_{16} + C18:1
	0.00	0.00	3.67
	0.00	0.00	5.61
	0.00	0.00	5.54
	0.00	0.00	2.44
	0.00	0.00	0.00
	0.00	0.00	0.00
	0.00	0.00	
	0.00	0.00	
	0.00	0.00	
	0.00	0.00	

24h	BODIPY FL C_{16}	BODIPY FL C_{16} + C16:0	BODIPY FL C_{16} + C18:1
	0.00	0.00	4.13
	0.00	0.00	4.79
	0.00	0.00	5.04
	0.00	0.00	9.72
	0.00	0.00	7.31
	0.00	0.00	4.31
	0.00	0.00	4.09
	0.00	0.00	
	0.00	0.00	
	0.00	0.00	

Raw data for Fig. 2B: Quantification of the total are of INS-1E cells covered by fluorescent puncta after treatment with C16:0 (500µM) and BODIPY FL C16 (400nM) alone or in combination with 250µM C18:1.

2h	BODIPY FL C_{16}	BODIPY FL C_{16} + C16:0
	0.00	1.22
	0.00	1.22
	0.00	2.79
	0.00	1.36
	0.00	4.65
	0.00	0.90
	0.00	0.69
	0.00	0.00
	0.00	0.00
	0.00	0.00

6h	BODIPY FL C_{16}	BODIPY FL C_{16} + C16:0
	0.00	2.15
	0.00	1.05
	0.00	4.10
	0.00	6.84
	0.00	4.34
	0.00	1.90
	0.00	6.30
	0.00	2.74
	0.00	1.91
	0.00	3.76
		2.08

24h	BODIPY FL C_{16}	BODIPY FL C_{16} + C16:0
	0.00	9.49
	0.00	11.67
	0.00	5.54
	0.00	13.84
	0.00	17.45
	0.00	8.92
	0.00	11.93
	0.00	15.46
	0.00	18.23
	0.00	12.69
	0.00	9.35
	0.00	10.02
		5.19

Raw data for Fig. 2C: Pearson correlation coefficient to demonstrate the co-localisation of BODIPY FL C16 with PLIN2 in EndoC-βH1 and INS-1E cells treated with C16:0 +/- C18:1.

EndoC-βH1 C16:0	INS-1E C16:0	INS-1E C16:0+C18:1
0.64	0.2	0.69
0.51	0.21	0.58
0.55	0.08	0.73
0.74	0.33	0.79
0.567	0.13	0.71
0.58	0.38	0.49

Raw data for Fig. 4C: Pearson correlation coefficient to demonstrate the co-localisation of BODIPY FL C16 and CellLight Golgi-RFP in EndoC-βH1 and INS-1E cells treated with C16:0 +/- C18:1.

INS-1 C16:0	EndoC-βH1 C16:0
0.64	0.26
0.57	0.3
0.49	0.01
0.68	0.23
0.67	0.3

Raw data for Fig. 5A-D: INS-1E cells were treated with either 250µM C16:0, 250µM C18:1, 250µM C19:0, 250µM C16:0 + 250µM C18:1, or 5µg/ml Tunicamycin for 6h or 16h. Total eIF2a, peIF2a, CHOP and GAPDH levels were analysed by Western blotting and quantified as peIF2α/total eIF2α ratio or CHOP/GAPDH ratio.

A. INS-1 peif2a/eif 6h

Vehicle	C16.0	C18.1	C16.0+C18.1	Tunicamycin
0.34648	0.359425	0.371968	0.246501	0.61561
0.116273	0.188032	0.100138	0.056933	0.35473
0.235421	0.305675	0.174448	0.090487	0.767011
0.231006	0.35988	0.352319	0.332568	0.809762

B. INS-1 peif2a/eif 16h

Vehicle	C16.0	C18.1	C16.0+C18.1	Tunicamycin
0.098459	0.119771	0.153359	0.115623	0.602362
0.079686	0.104778	0.110982	0.084102	0.563482
0.377515	0.377772	0.223857	0.240524	1.248146
0.346243	0.448156	0.704467	0.560031	0.994813
0.586535	0.734115	0.58094	0.560343	1.360326

C. INS-1 CHOP 6h

Vehicle	C16.0	C18.1	C16.0+C18.1	Tunicamycin
0.284053	0.575032	0.429228	0.471097	1.267003
0.498714	0.722078	0.710788	0.513608	0.840967
0.253836	0.266555	0.195764	0.219375	0.442682
0.329529	0.235861	0.322306	0.435529	0.682446

D. INS-1 CHOP 16h

Vehicle	C16.0	C18.1	C16.0+C18.1	Tunicamycin
0.210109	0.240953	0.326351	0.207606	0.456582
0.160895	0.494086	0.422976	0.436821	1.034611
0.138276	0.181319	0.19816	0.1734	0.807969
0.341225	0.311787	0.35772	0.430945	0.683465

Raw data for Fig. 5E-H: EndoC-βH1 cells were treated with either 250µM C16:0, 250µM C18:1, 250µM C19:0, 250µM C16:0 + 250µM C18:1, or 5µg/ml Tunicamycin for 6h or 16h. Total eIF2a, peIF2a, CHOP and Western blotting and quantified as peIF2α/total eIF2α ratio or CHOP/GAPDH ratio.

E. EndoC-BH1 peif2a/eif 6h

Vehicle	C16.0	C18.1	C16.0+C18.1	C19:0
0.73787	0.61552	0.213996	0.225601	0.43138
0.710839	0.572077	0.293398	0.452821	0.381116
0.763446	0.411326	0.318784	0.613437	0.315201

F. EndoC-BH1 peif2a/eif 18h

Vehicle	C16.0	C18.1	C16.0+C18.1	C19:0	Tunicamycin
0.850501	0.585974	0.349128	0.872201	0.86256	1.202224
0.852473	0.353947	0.186298	0.164667	0.194146	0.983159
0.492459	0.338988	0.329944	0.311498	0.189645	0.706628

G. EndoC-BH1 CHOP 6h

Vehicle	C16.0	C18.1	C16.0+C18.1	C19:0
0.220275	0.206575	0.154855	0.014336	0.118543
0.370521	0.402648	0.338492	0.245298	0.242491
0.355876	0.134027	0.264036	0.449371	0.293785

H. EndoC-BH1 CHOP 18h

Vehicle	C16.0	C18.1	C16.0+C18.1	C19:0	Tunicamycin
0.120117	0.225966	0.204019	0.149214	0.143958	0.305506
0.336496	0.22395	0.253194	0.075316	0.166249	1.904165
0.541734	0.362446	0.218513	0.253294	0.38567	1.080142
